# Supplementary figures and images for: Transcriptional Repression of reaper by Stand Still Safeguards Female Germline Development in Drosophila
Source: bioRxiv. 2025 Jun 3:2025.05.17.654630. Preprint. [Version 2] doi: 10.1101/2025.05.17.654630 (PMC12157695; doi:10.1101/2025.05.17.654630)

# Figure S1

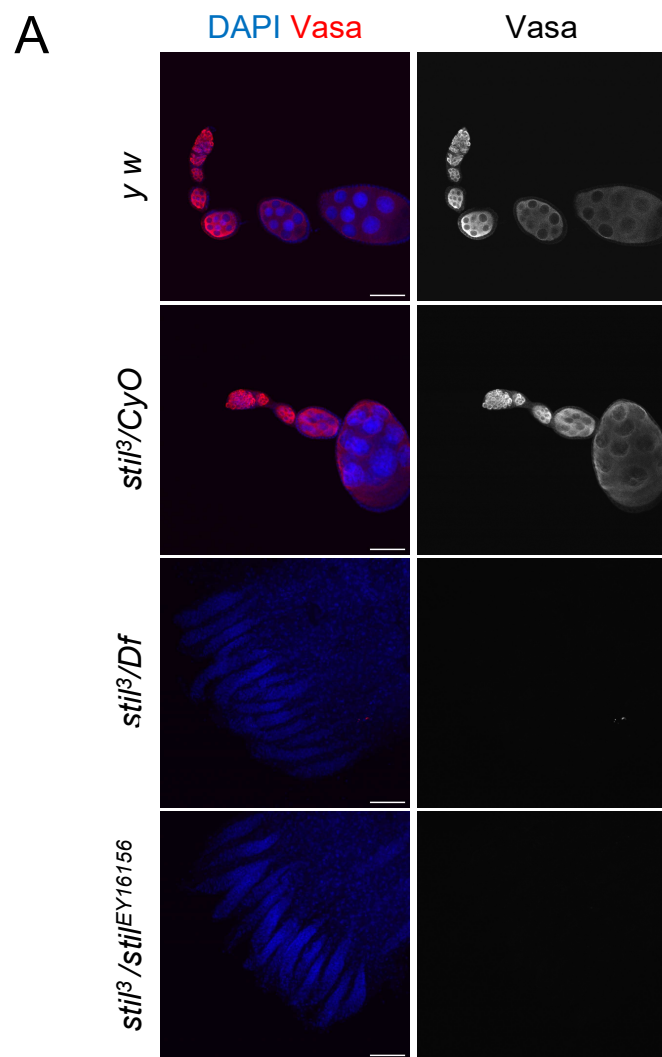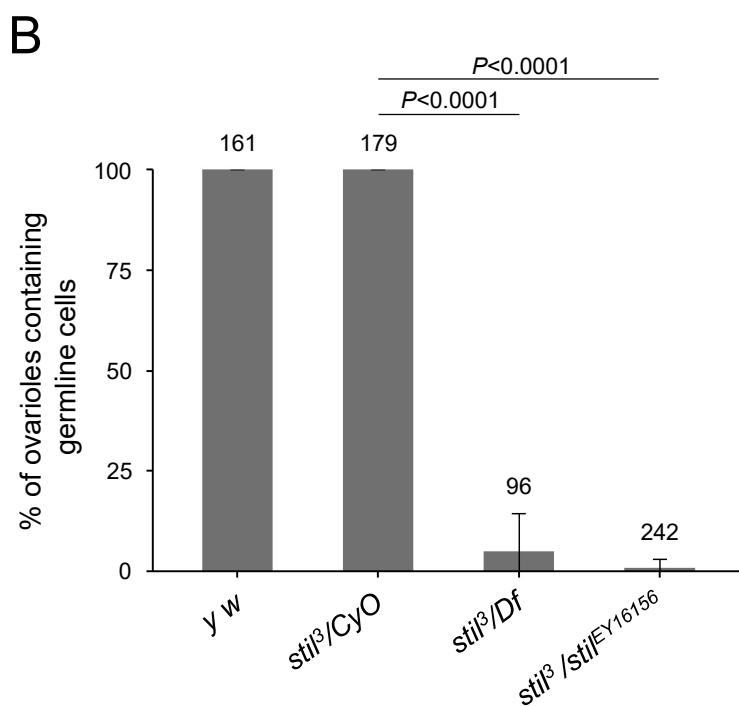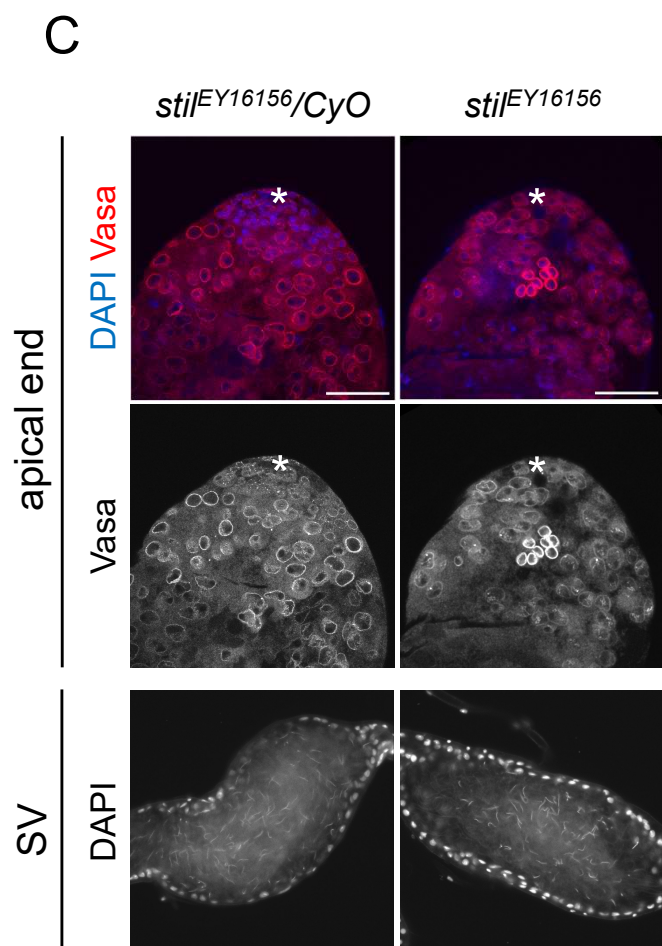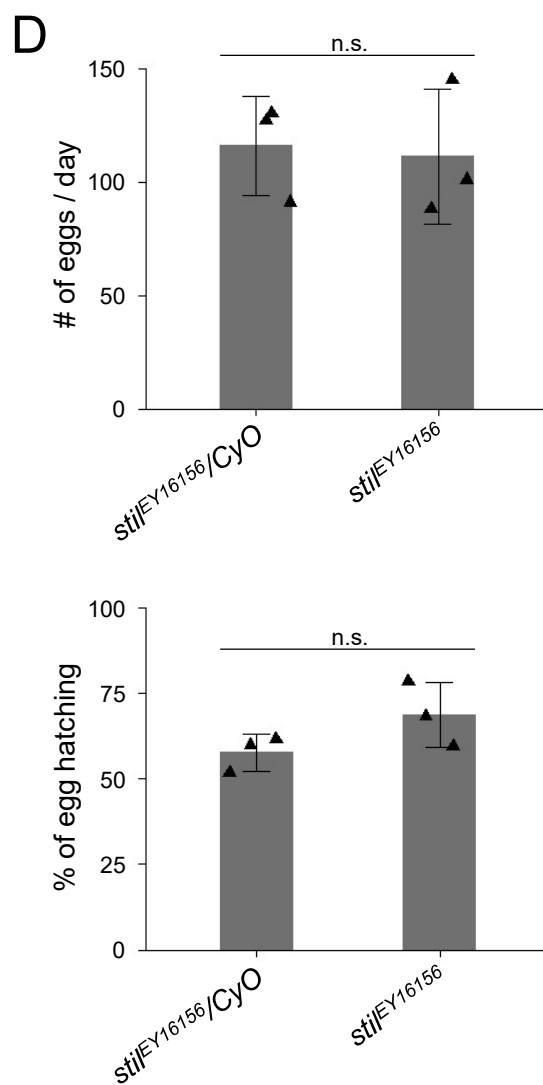

Figure S2

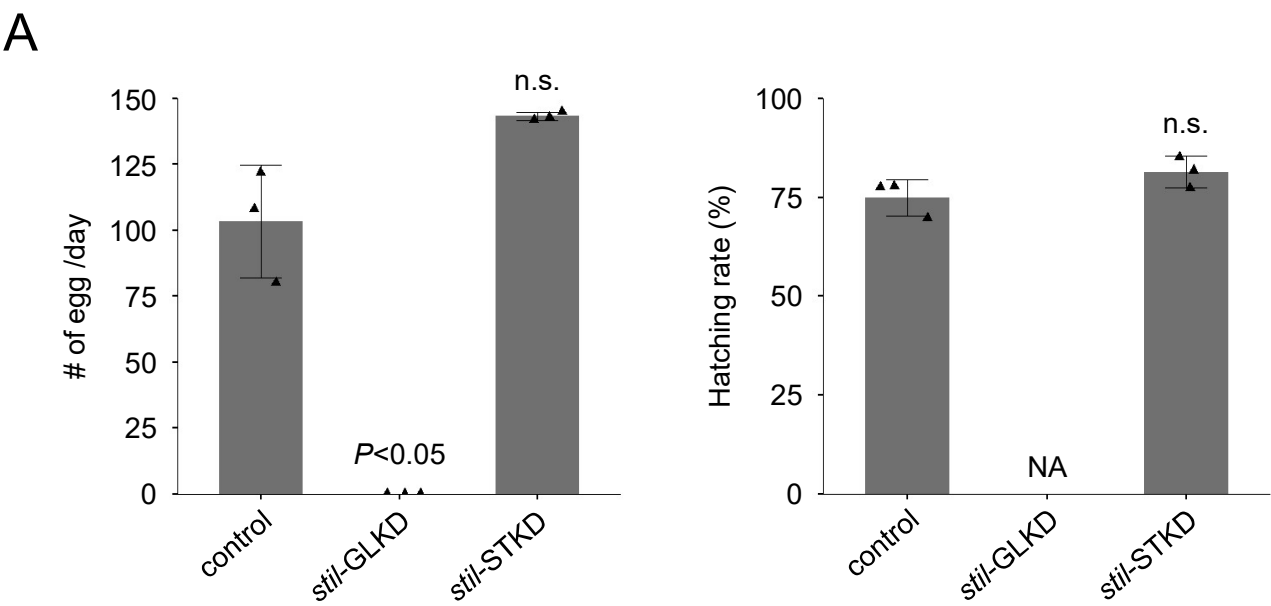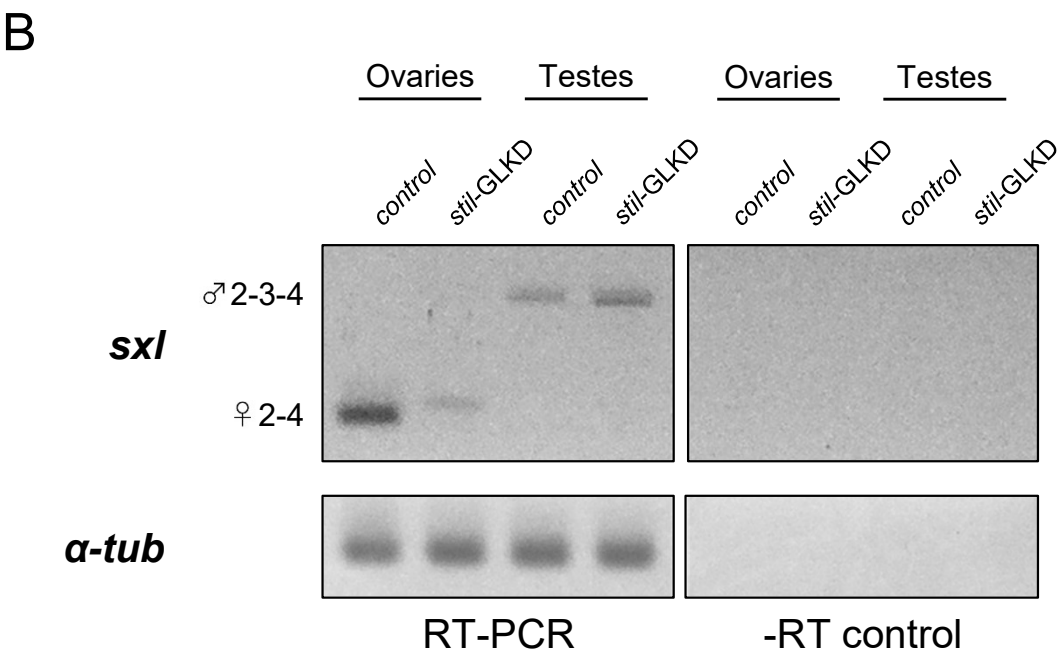

Figure S3

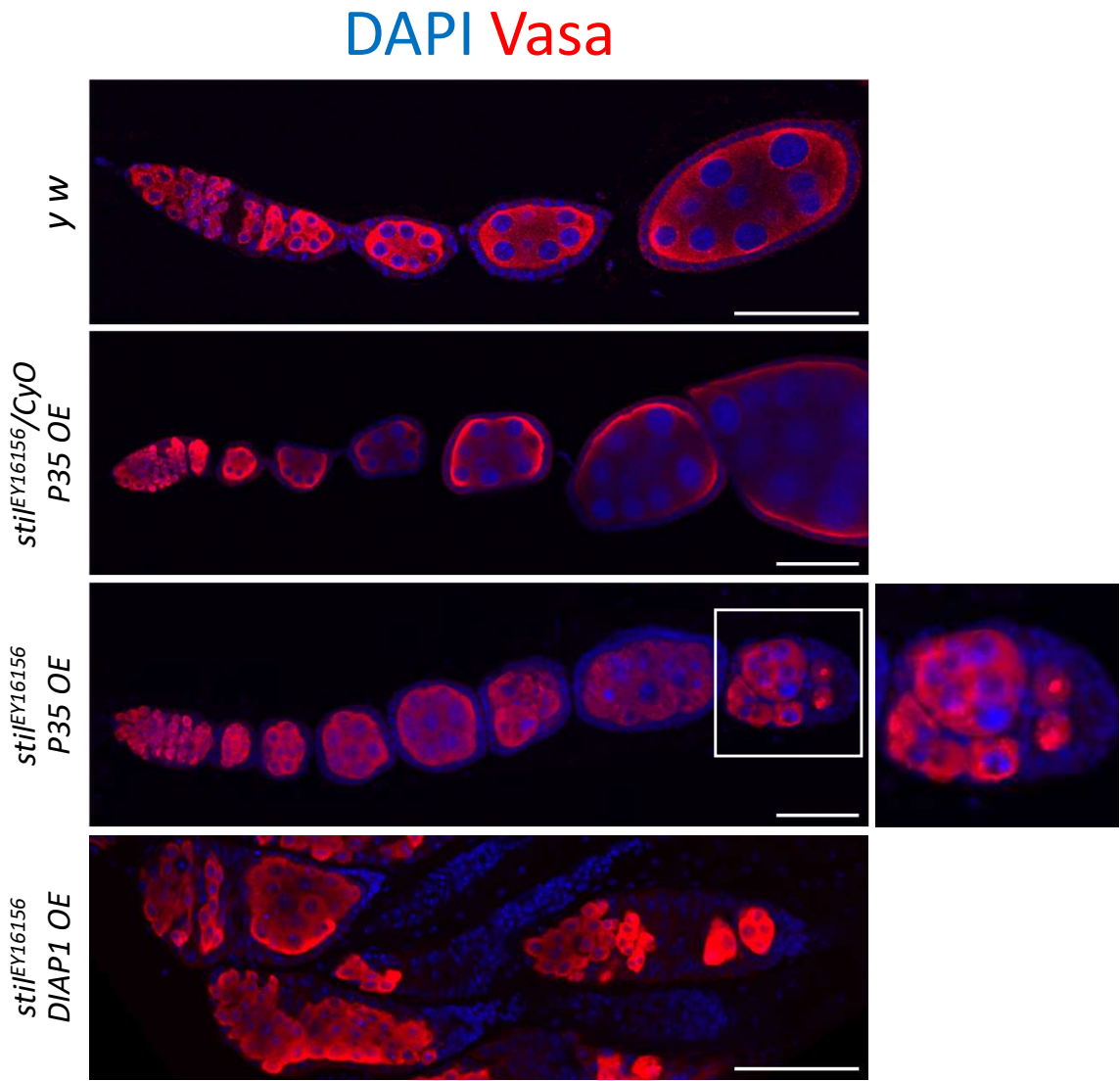

Figure S4

A

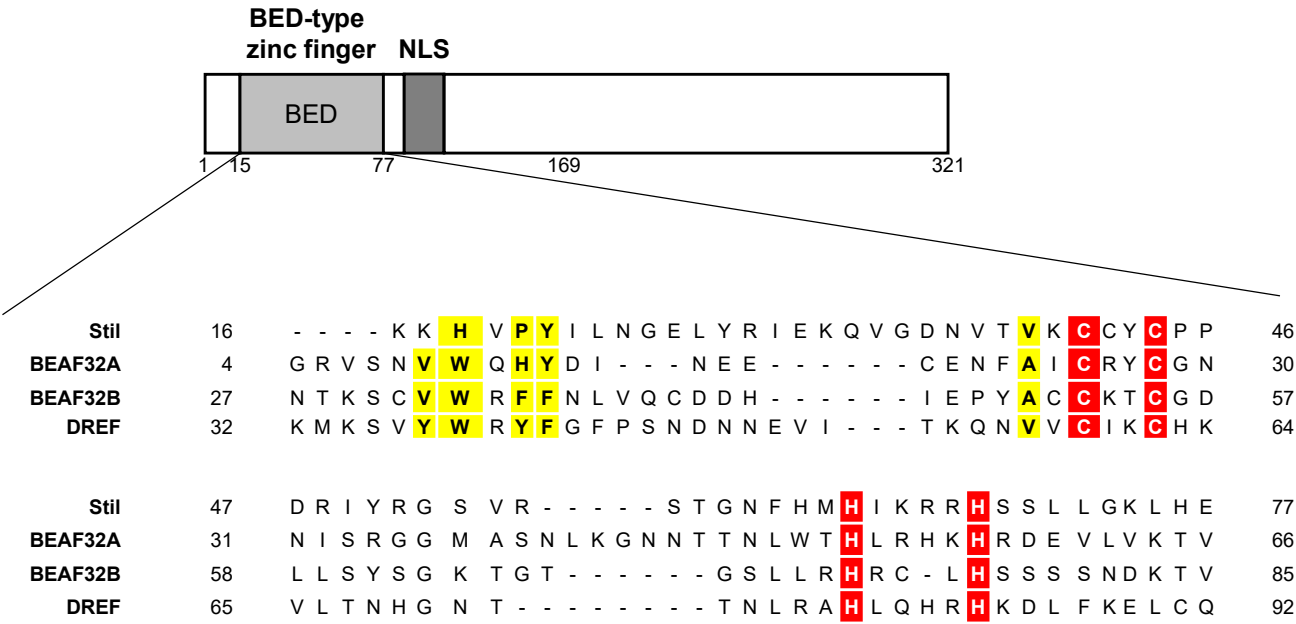

B

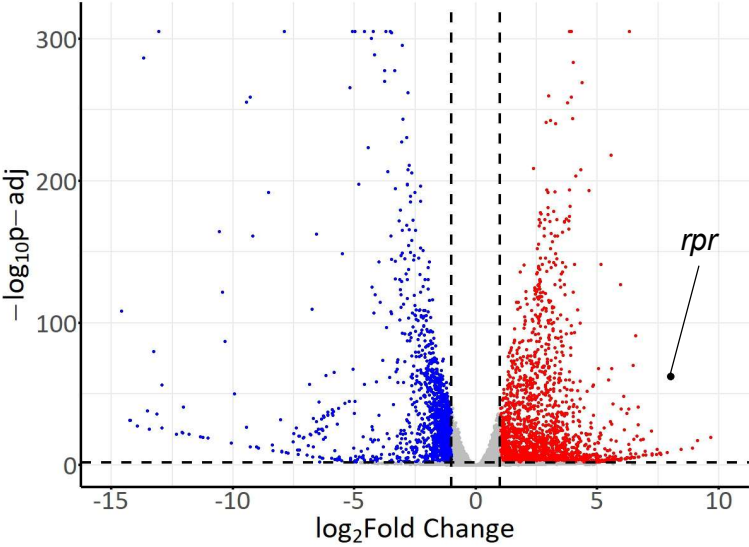

# Figure S5

**A**

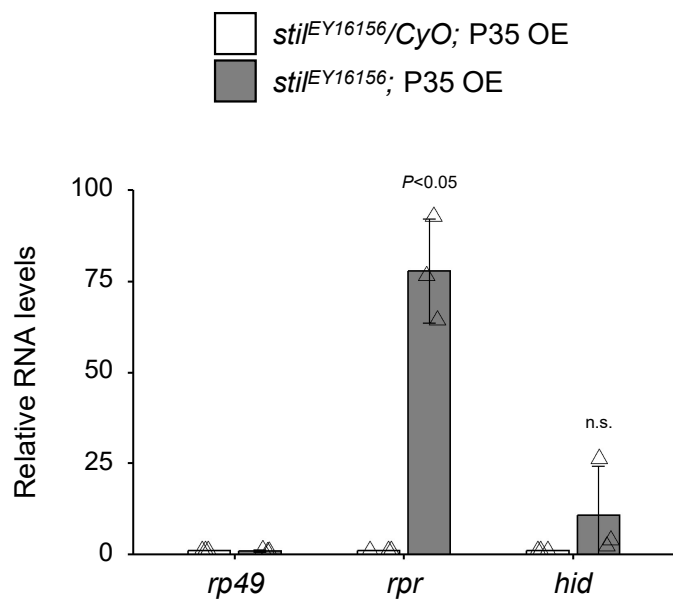

**C**

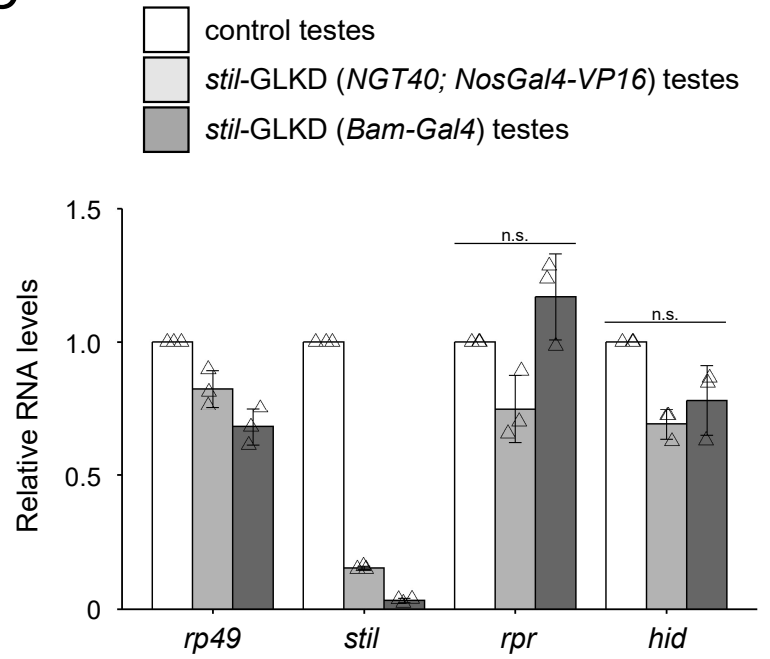

**B**

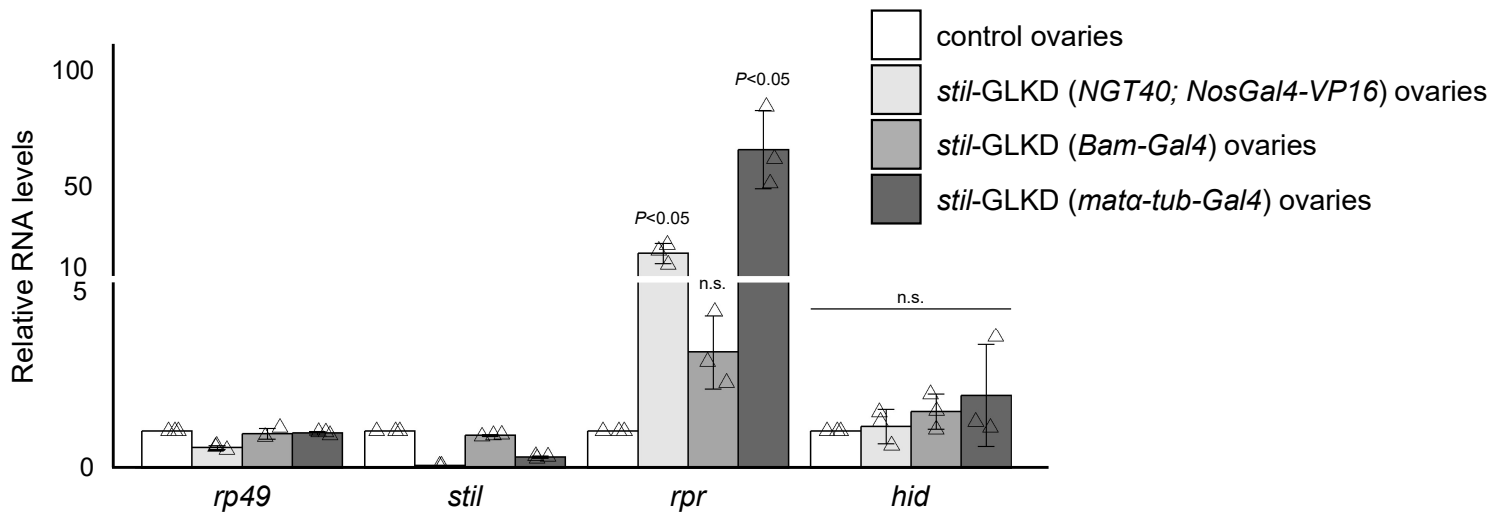

**D**

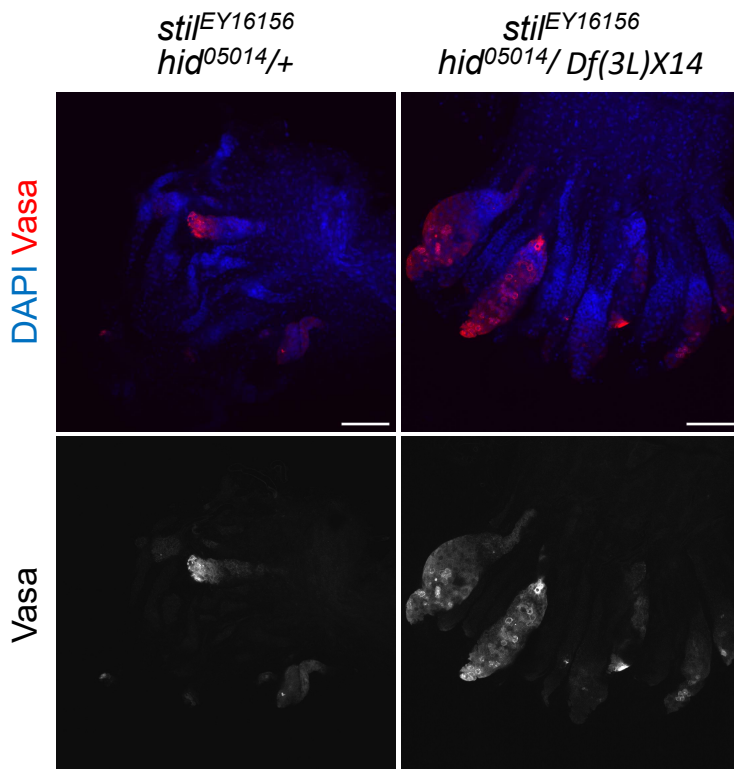

**E**

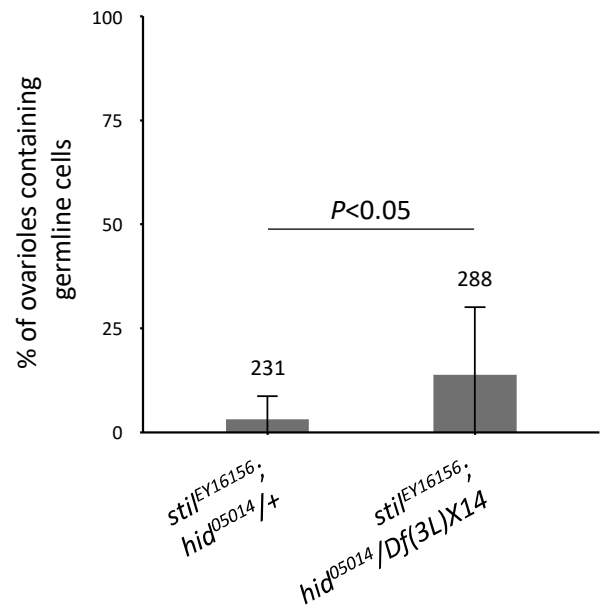

# Figure S6

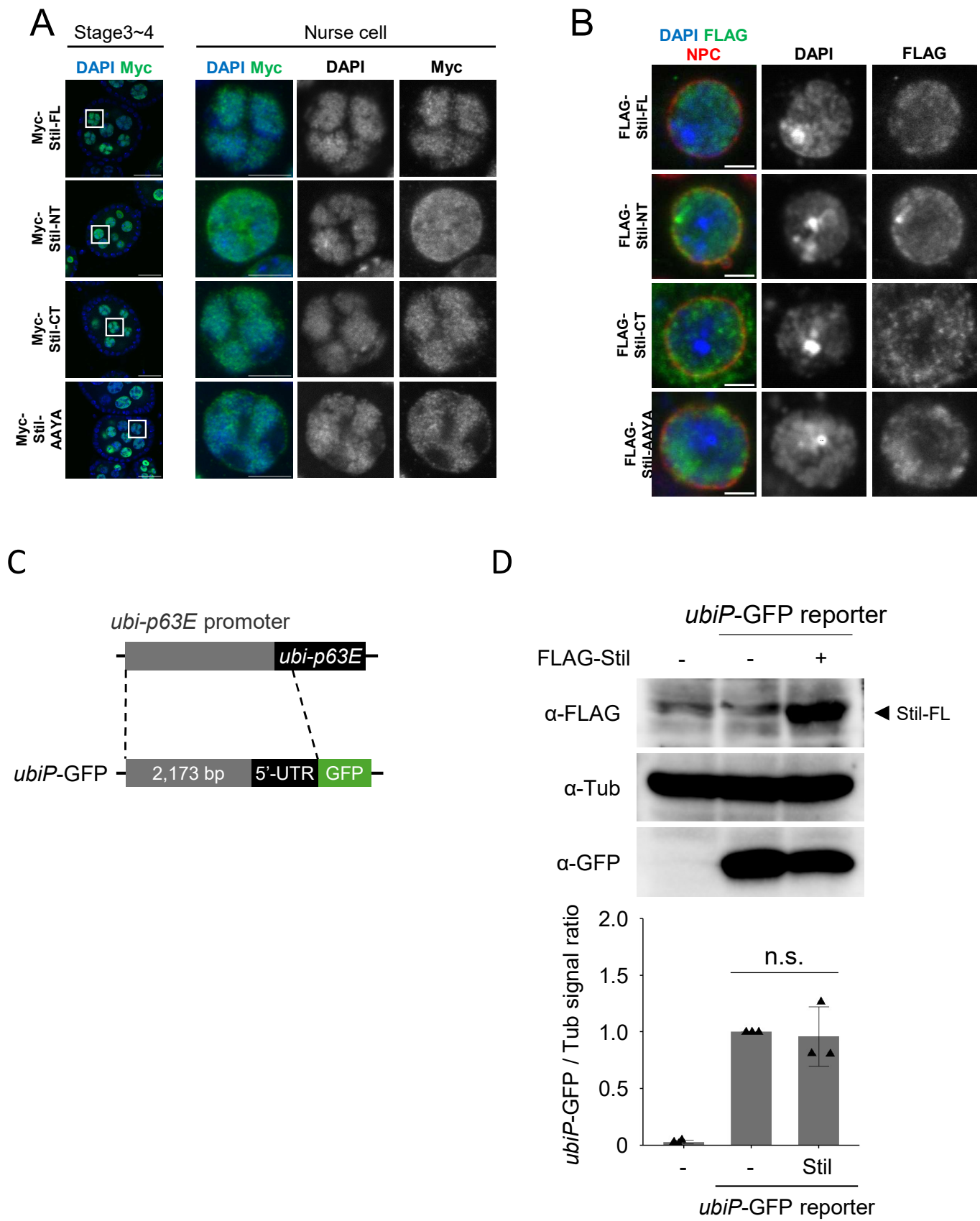

Supplement: Supplement 4 — Figure S1. stil function is indispensable in female germline cells not male germline cells (A) Immunostaining of ovaries from y w, stil3/CyO, stil3/Df, and stil3/stilEY16156 with antibody against Vasa (red) with DAPI (blue). Scale bar: 50 μm. (B) Quantification of the percentage of germline cell-containing ovarioles in 2–3 days old females. (C) Immunostaining of the apical end of testes from stilEY16156/CyO and stilEY16156 with anti-Vasa antibody (red) and DAPI (blue) (top panels). Seminal vesicles (SV) harboring sperms are also stained with DAPI (blue) (bottom panels). Asterisk denotes the apex of testis. Scale bar: 50μm (apical end of testes, top) and 20 μm (SV, bottom). (D) The numbers of egg laying and hatching rate. Daily egg laying by three y w females mated with three males of the indicated genotypes are shown (n=3). The number of ovarioles assessed is noted above each bar. Error bar indicates s.d. Figure S2. Germline knockdown of stil, but not somatic knockdown, specifically disrupts egg development (A) Analysis of egg laying and hatching rates. The number of laid eggs and their hatching rates are measured daily for three females of the indicated genotypes: control, stil-germline knockdown (stil-GLKD) driven by NGT40; NosGal4-VP16, and stil-somatic knockdown (stil-STKD) driven by tj-Gal4, each mated with three y w males (n = 3). Error bars indicate standard deviation (s.d.). (B) RT-PCR analysis of sxl and the control, α-tub transcripts in ovaries and testes from control and stil-GLKD flies, respectively. Figure S3. Egg development is disrupted at the mid-stage of oogenesis in stil mutants rescued by P35 or DIAP1 Immunostaining of ovarioles from y w, stilEY16156/CyO; P35 OE (NGT40; NosGal4-VP16> P35), stilEY16156; P35 OE and stilEY16156; DIAP1 OE (NGT40; NosGal4-VP16> DIAP1) flies with antibody against Vasa (red) and DAPI (blue). The enlarged image highlights a degenerated egg chamber at the mid-stage of oogenesis in stilEY16156; P35 OE. Scale bar: 50 μm. Fig [file media-4.pdf]
